# Supplementary material for: Targeting neovascularization and respiration of tumor grafts grown on chick embryo chorioallantoic membranes
Source: PLoS One. 2021 May 17;16(5):e0251765. doi: 10.1371/journal.pone.0251765 (PMC8128225; doi:10.1371/journal.pone.0251765)
Supplement: S1 Raw images — (PDF) [file pone.0251765.s001.pdf]

**S1 raw images**

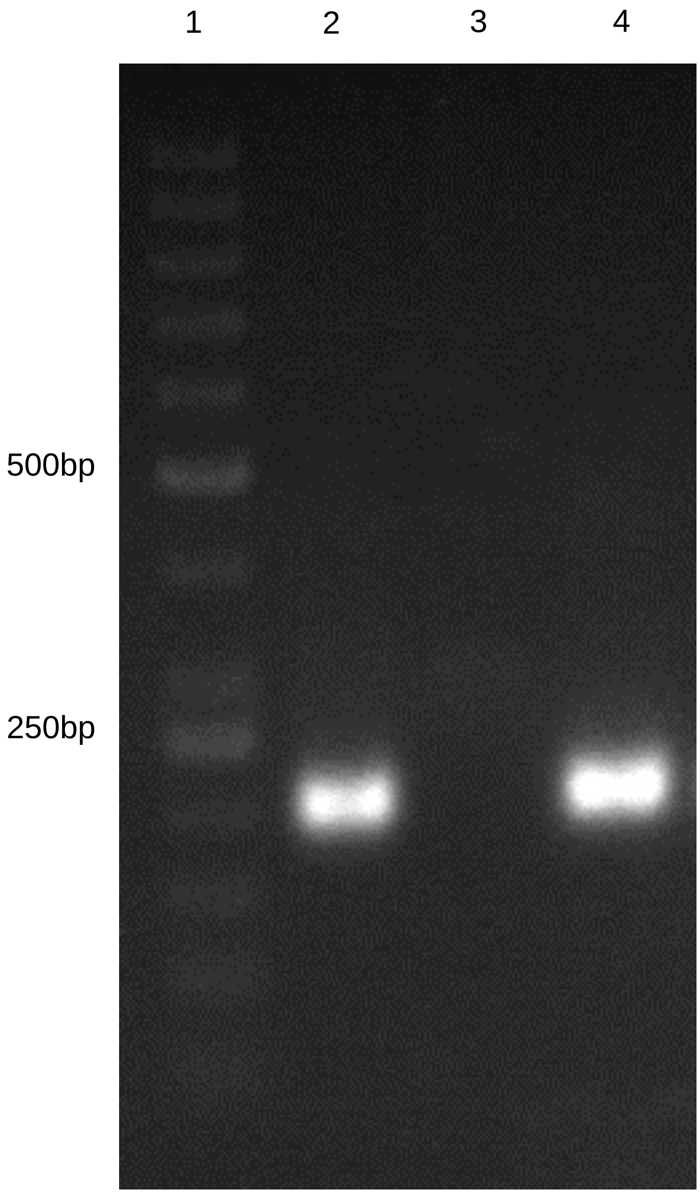


Loading: PCR product using primers against human mitochondrial D-Loop, DNA used as indicated below
1: DNA ladder plus 50bp
2: DNA MDA-MB231
3: DNA chicken liver
4: DNA mix MDA-MB231 and CAM

PCR products were visualized by Gel Doc gel imaging system from BioRad and saved as jpg.

This blot was used creating figure panel 5b left

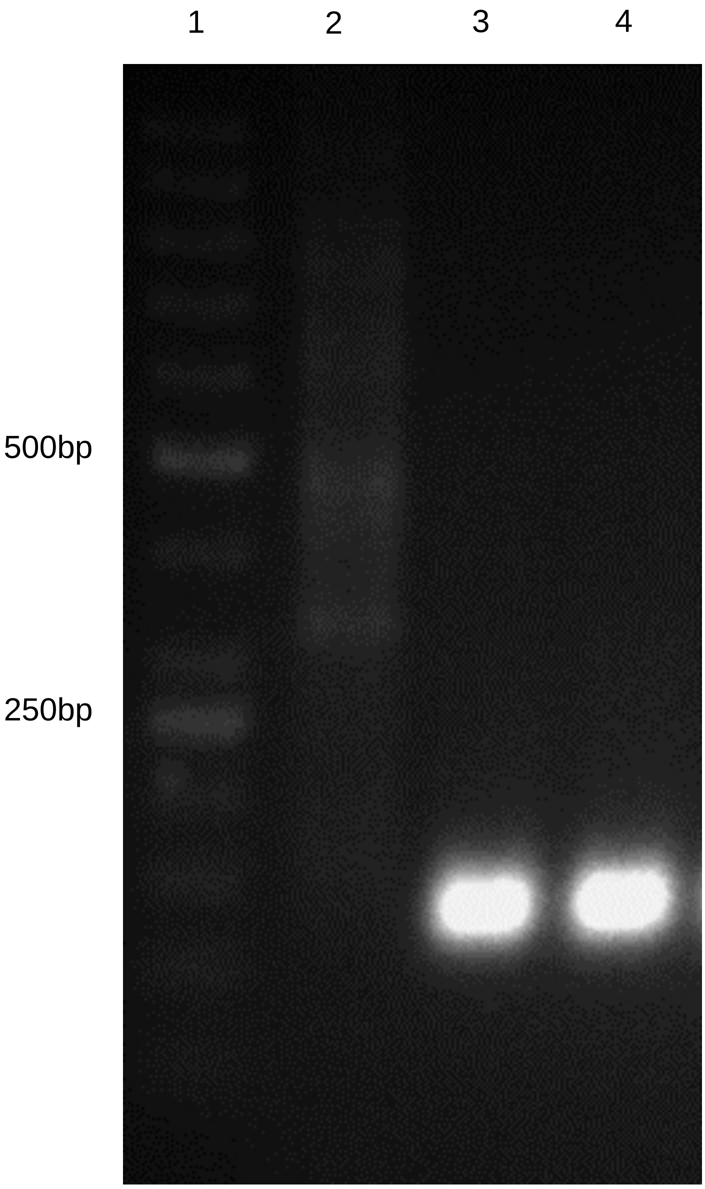


Loading: PCR product using primers against chicken mitochondrial D-Loop, DNA used as indicated below
1: DNA ladder plus 50bp
2: DNA MDA-MB231
3: DNA chicken liver
4: DNA mix MDA-MB231 and CAM

PCR products were visualized by Gel Doc gel imaging system from BioRad and saved as jpg.

This blot was used creating figure panel 5b right
