# Supplementary material for: Targeting neovascularization and respiration of tumor grafts grown on chick embryo chorioallantoic membranes
Source: PLoS One. 2021 May 17;16(5):e0251765. doi: 10.1371/journal.pone.0251765 (PMC8128225; doi:10.1371/journal.pone.0251765)
Supplement: S1 Table — (PDF) [file pone.0251765.s002.pdf]

S1 Table

**Name**	**Sequence**
Human specific Fwd primer	5´CTAAATAGCCCACACGTTCC 3´
Human specific Rev primer	5´ TAGGATGAGGCAGGAATCAA 3´
Human Probe	5´ FAM-TCACGATGGATCACAGGTC-*BHQ1* 3´
Chicken specific Fwd primer	5´ TACTTCATGACCAGTCTCAGG 3´
Chicken specific Rev primer	5´ AGTTCAGGAGTTATGCATGG 3
Chicken Probe	5´ YY-ACCGTACCTCTGGTTCCTC-*BHQ1* 3´

S1 Table. **Primer/probe sequences used in ddPCR**. Fluorophores (underlined): FAM = fluorescein amidite, YY = Yakima Yellow; Quencher (italic): BHQ1 = black hole quencher 1. Primes and probes where ordered from Microsynth AG (Balgach, Switzerland).

ddPCR conditions

The primer probe pairs in Suppl. Table 1 were used in a single reaction together with 1ng/ul of zone 0-3 extracted DNA and 10ul of 2x supermix (ddPCR™ Supermix for Probes #186-3010: Bio-Rad), in a 20ul reaction. PCR was carried out at 95 °C × 10 min (1 cycle), 94 °C × 30 s and 60.5 °C × 30 s (40 cycles), 98 °C × 10 min (1 cycle), and 12 °C hold
