# Supplementary material for: Targeting neovascularization and respiration of tumor grafts grown on chick embryo chorioallantoic membranes
Source: PLoS One. 2021 May 17;16(5):e0251765. doi: 10.1371/journal.pone.0251765 (PMC8128225; doi:10.1371/journal.pone.0251765)
Supplement: S2 Table — (PDF) [file pone.0251765.s003.pdf]

S2 Table

**Name**	**Sequence**
Human D-Loop forward	5'-CTAAATAGCCCACACGTTCC-3'
Human D-Loop reverse	5'-TTGATTCCTGCCTCATCCTA-3'
Canine D-Loop forward	5'-GCCACGGCATTCATAGGT-3'
Canine D-Loop reverse	5'-TTTGGATTGCTTTATCAACTGG-3'
Chicken D-Loop forward	5'-TACTTCATGACCAGTCTCAGG-3'
Chicken D-Loop reverse	5'-AGTTCAGGAGTTATGCATGG-3'
Human MCT1forward	5'-GTGGCTCAGCTCCGTATTGT-3'
Human MCT1 reverse	5'-GAGCCGACCTAAAAGTGGTG-3'
Human MCT4 forward	5'-GCATCTTCTTTGGCATCTCC-3'
Human MCT4 reverse	5'-GCTCTTTGGGCTTCTTCCTAA-3'
Human β-actin forward	5'-TGGCATCCACGAAACTACCT-3'
Human β–actin reverse	5'-CTCGTCATACTCCTGCTTGCT-3'

S2 Table. Primer used in SYBR Green qPCR.

SYBR Green qPCR

PCR was carried out at 95 °C × 10 min (1 cycle), 95 °C × 30 s and 60 °C × 30 s (40 cycles), 95 °C × 15sec and 60°C x 15sec (2 cycles).
