# Supplementary material for: Targeting neovascularization and respiration of tumor grafts grown on chick embryo chorioallantoic membranes
Source: PLoS One. 2021 May 17;16(5):e0251765. doi: 10.1371/journal.pone.0251765 (PMC8128225; doi:10.1371/journal.pone.0251765)
Supplement: S1 Fig — (PDF) [file pone.0251765.s004.pdf]

S1 Fig

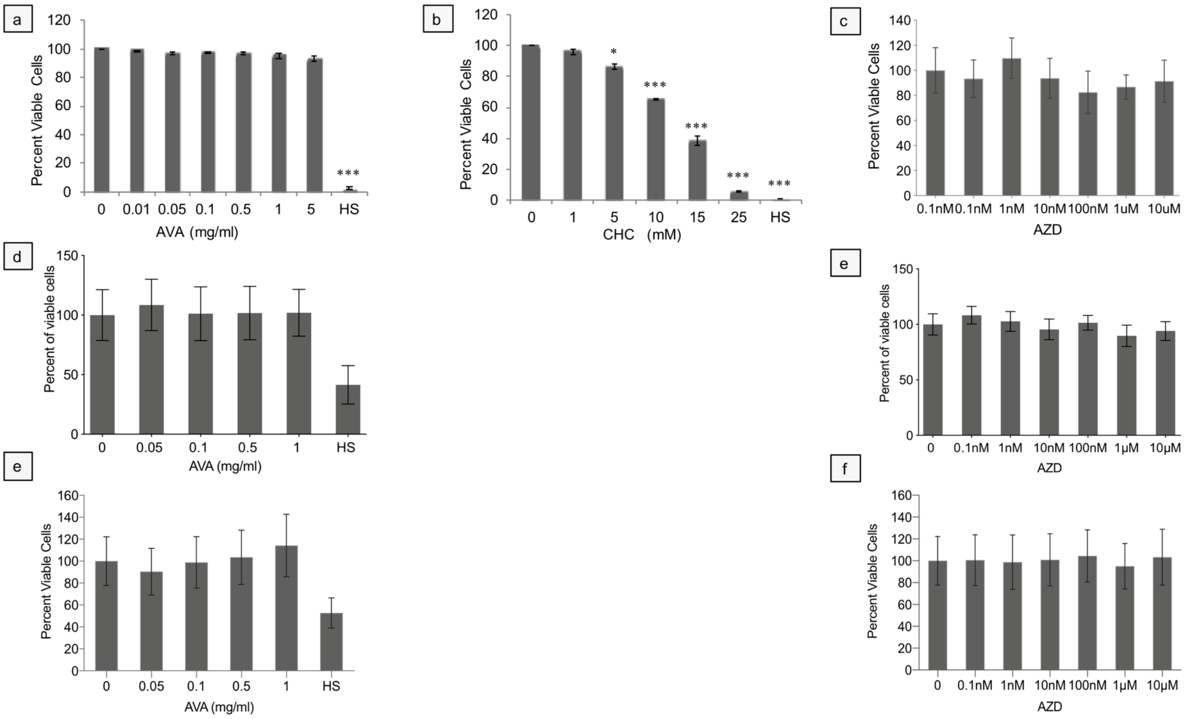

S1 Fig. In vitro drug toxicity assay. Use of drugs at non-toxic concentrations was ascertained by applying i) increasing concentrations of AVA, CHC and AZD (AVA = Avastin, CHC = α-cyano-4-hydroxycinnamic acid, AZD = AZD3965) to cultured cells (*in vitro* toxicity), and ii) doses of the respective compound, corresponding to validated safe-usage applications with human patients, onto the CAM on d10 of embryo development (*in vivo* toxicity).

Regarding the *in vitro* toxicity of AVA, CHC and AZD, viability of cells was recorded by Trypan Blue exclusion assays in response to increasing concentrations of AVA, CHC and AZD. Maximal cell death was induced by 0.1M NaCl solution (i.e. HS = high salt) and all treatments were compared to non-treated CTRL (0). No cytotoxicity could be seen with AVA concentrations 0.01-5 mg/ml for human U87 (a) and also for canine 17CM98 (d) and canine D17(f) (AVA concentration 0.05-1mg/ml) , whereas CHC imparted significant cell death in U87 cells at concentrations ≥5 mmol (b). Also with AZD no cytotoxicity could be seen by the applied concentration range from 0.1nM to 10μM in human U87 (c), canine 17CM98 (e) and D17 (g). Data are means ± SEM, n=3 for U87 (a and b) and n=5 for (c); n=9 for (d), n=8 for (e); n=9 for (f) and (g). Statistical differences against CTRL were tested with one-way ANOVA for (a) and (b), and significance was accepted at $p<0.05$. * = $p<0.05$, *** = $p<0.001$. Statistical analysis was done by using Kruskal-Wallis for (c) to (g), ns.

Regarding *in vivo* assays, embryo weight, taken from published chick embryo development charts [65] was considered and 10mg/kg AVA, 60mg/kg CHC and 2.5μM/egg AZD was applied onto CAMs. 10mg/kg AVA is an effective concentration in human application (Roche), while 60mg/kg CHC was used since its corresponding molarity (assuming 1kg ≅ 1l) of 0.317 mM lies well within the non-toxic range of U87 *in vitro* toxicity data (panel b above) and since 80-100mg/kg CHC was completely inhibitory in previous bioassays [66]. Thus, to study the effect of AVA in combination as well, CHC concentration was reduced to 60mg/kg. CHC was later replaced by AZD, since AZD is a more specific MCT1-inhibitor, especially up to 10μM. As control for AVA, CHC and AZD, equal volumes of PBS and DMSO were applied to separate CAMs, respectively. No fatalities or toxic effects on embryos (i.e. hemorrhage, hyperemia or coagulation at application site) were observed by these drug doses by d14 of development. Thus, these concentrations were also used in experiments with tumor explants (see text).
