# Supplementary material for: Targeting neovascularization and respiration of tumor grafts grown on chick embryo chorioallantoic membranes
Source: PLoS One. 2021 May 17;16(5):e0251765. doi: 10.1371/journal.pone.0251765 (PMC8128225; doi:10.1371/journal.pone.0251765)
Supplement: S2 Fig — (PDF) [file pone.0251765.s005.pdf]

S2 Fig

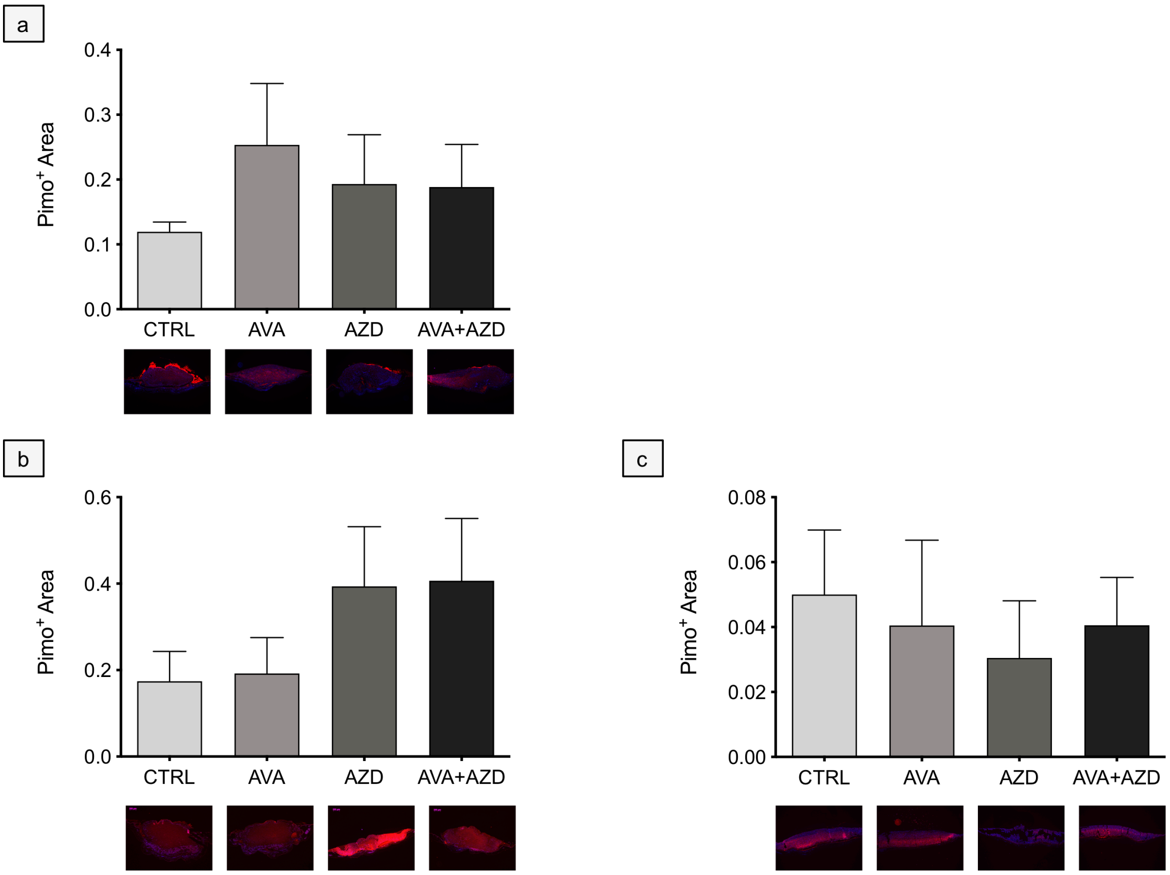

S2 Fig. **Tumor tissue hypoxia on paraffin section - pimonidazole.** Tumor explants of (a) human glioblastoma U87, (b) canine oral melanoma 17CM98 and (c) canine osteosarcoma were treated as indicated: CTRL (PBS, 50µl, i.v. injected), AVA (Avastin®, i.v. injected, 10mg/kg), AZD (AZD3965, i.v. injected, 2.5µM/egg) and AVA+AZD combination. Pimonidazole (60mg/kg) was injected i.v. 30min prior to euthanasia. Tumor explants were collected and paraffin sections were prepared and subjected to immunofluorescence staining. No differences between the different treatments in the differenct cell lines could be found. Data are means ± SEM. (a) n=5; (b) n=4 and (c) n= 6. Statistical analysis: one-way ANOVA, ns.

Immunofluorescence (IF) staining on paraffin sections was done for human U87 and canine 17CM98 and D17 explants to detect tissue hypoxia. Pimonidazole was injected i.v. (60mg/kg) into tumor-bearing embryos on d14, 20min prior to tumor harvest. Tumors were then fixed in 4% paraformaldehyde for 24h and embedded in paraffin. Finally, 2µM sections were prepared. Paraffin slides were deparaffinized and rehydrated, followed by tissue antigen retrieval procedure using sodium citrate buffer. Blocking was performed using 5% goat serum in PBS, after that, sections were incubated with PAb2627(AP) Rabbit Antisera (Hypoxyprobe^{TM}) overnight at 4°C (1:200 in 5% NGS/PBS + 0.1% Tween20). Cy3 goat anti rabbit antibody was used with 1:200 dilution mixed with 100nM DAPI, incubated 60min at RT. Slides were mounted with ProLongTM Gold antifade reagent and sealed with nail polish. Image

acquisition was done using identical microscope, camera and acquisition settings to quantify staining intensities and stained area with MCID software. The pimonidazole positive area in relation to the whole tumor area as proxy of the extent of tumor tissue hypoxia was determined.
