# Supplementary material for: Targeting neovascularization and respiration of tumor grafts grown on chick embryo chorioallantoic membranes
Source: PLoS One. 2021 May 17;16(5):e0251765. doi: 10.1371/journal.pone.0251765 (PMC8128225; doi:10.1371/journal.pone.0251765)
Supplement: S3 Fig — (PDF) [file pone.0251765.s006.pdf]

S3 Fig

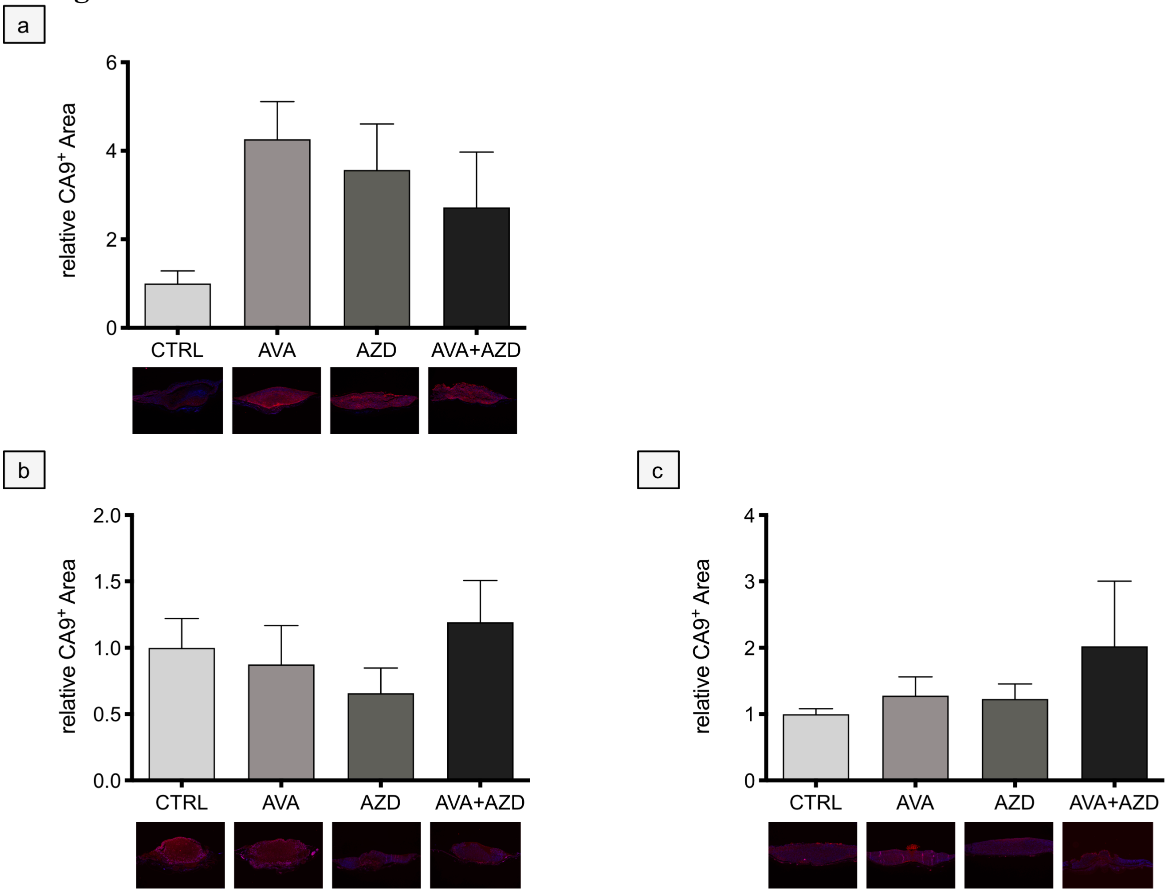

S3 Fig. Tumor tissue hypoxia on paraffin section – carbonic anhydrase IX (CA IX). Tumor explants of (a) human glioblastoma U87, (b) canine oral melanoma 17CM98 and (c) canine osteosarcoma were treated as indicated: CTRL (PBS, 50µl, i.v. injected), AVA (Avastin®, i.v. injected, 10mg/kg), AZD (AZD3965, i.v. injected, 2.5µM/egg) and AVA+AZD combination. Tumor explants were collected and paraffin sections were prepared and subjected to immunofluorescence staining for CA IX. No differences between the different treatments in the differenct cell lines could be found. Data are means ± SEM. (a) n=5; (b) n=5 and (c) n= 6. Statistical analysis: one-way ANOVA, ns.

Immunofluorescence (IF) staining on paraffin sections was done for human U87 and canine 17CM98 and D17 explants to detect tissue hypoxia. Tumors were fixed in 4% paraformaldehyde for 24h and embedded in paraffin. Finally, 2µM sections were prepared. Paraffin slides were deparaffinized and rehydrated, followed by tissue antigen retrieval procedure using sodium citrate buffer. Blocking was performed using 5% goat serum in PBS, after that, sections were incubated with rabbit CA IX antibody (NB100-417) overnight at 4°C (1:1000 in 5% NGS/PBS + 0.1% Tween20). Cy3 goat anti rabbit antibody was used with 1:200 dilution mixed with 100nM DAPI, incubated 60min at RT. Slides were mounted with ProLongTM Gold antifade reagent and sealed with nail polish. Image acquisition was done using identical microscope, camera and acquisition settings to quantify staining intensities and stained area with MCID software. The pimonidazole positive area in relation to the whole tumor area as proxy of the extent of tumor tissue hypoxia was determined.
