# Supplementary material for: Targeting neovascularization and respiration of tumor grafts grown on chick embryo chorioallantoic membranes
Source: PLoS One. 2021 May 17;16(5):e0251765. doi: 10.1371/journal.pone.0251765 (PMC8128225; doi:10.1371/journal.pone.0251765)
Supplement: S4 Fig — (PDF) [file pone.0251765.s007.pdf]

S4 Fig

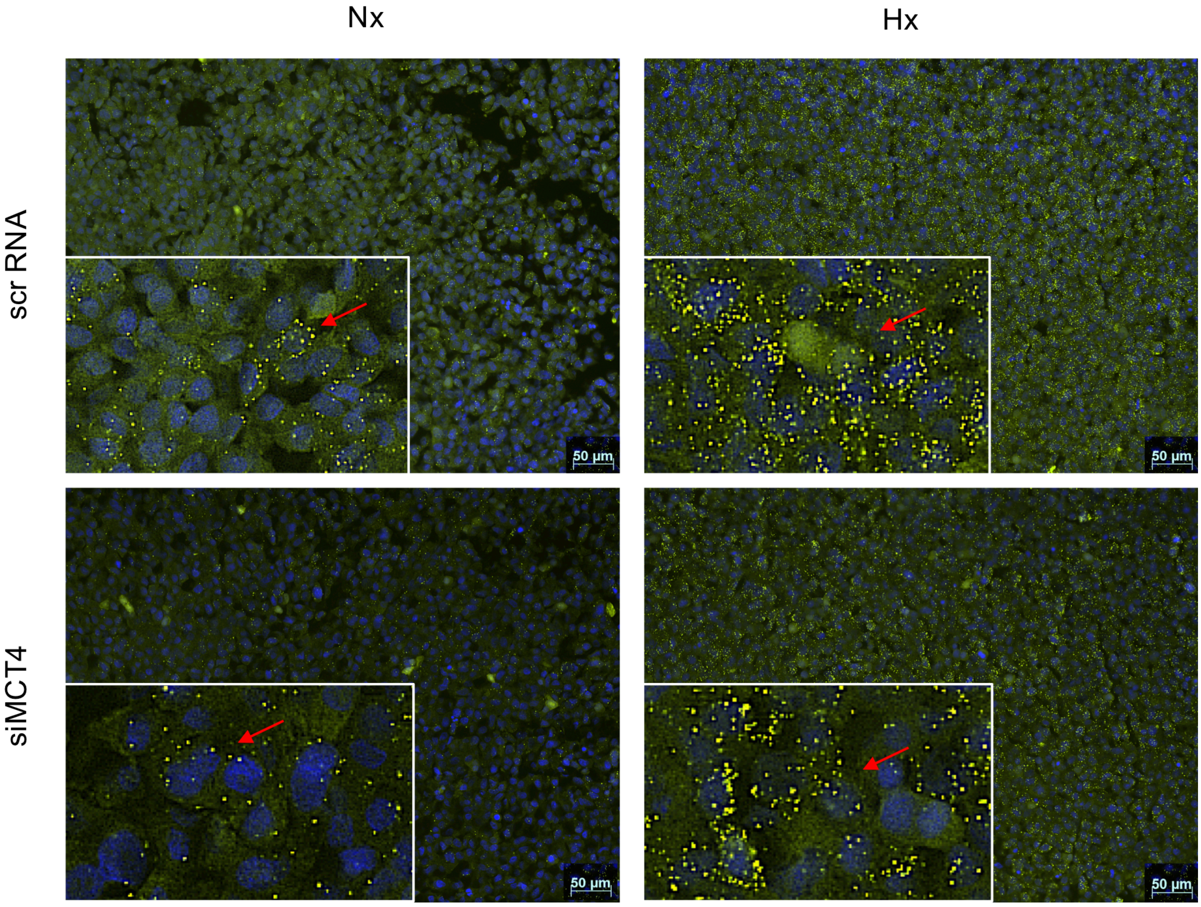

S4 Fig . Canine MCT4 staining. In situ hybridization staining with RNAscope® technology of specific MCT expression of canine MCT4. 17CM98 canine oral melanoma cells were transfected with siRNA against canine MCT4 using Lipofectamine 2000. Following transfection, cells were incubated in air (Nx) or 0.2% O_2 (Hx) for 16h. Positive signals appeared as dots (red arrows). Cells (scr and siMCT4) exposed to hypoxic conditions showed an increased RNA expression of MCT4. In the cells with RNA-based knockdown of canine MCT4 a reduced staining to fewer remaining signals could be observed in normoxic as well as hypoxic cells compared with scr RNA control. Scale bar = 50µm.
