# Supplementary material for: Targeting neovascularization and respiration of tumor grafts grown on chick embryo chorioallantoic membranes
Source: PLoS One. 2021 May 17;16(5):e0251765. doi: 10.1371/journal.pone.0251765 (PMC8128225; doi:10.1371/journal.pone.0251765)
Supplement: S5 Fig — (PDF) [file pone.0251765.s008.pdf]

S5 Fig

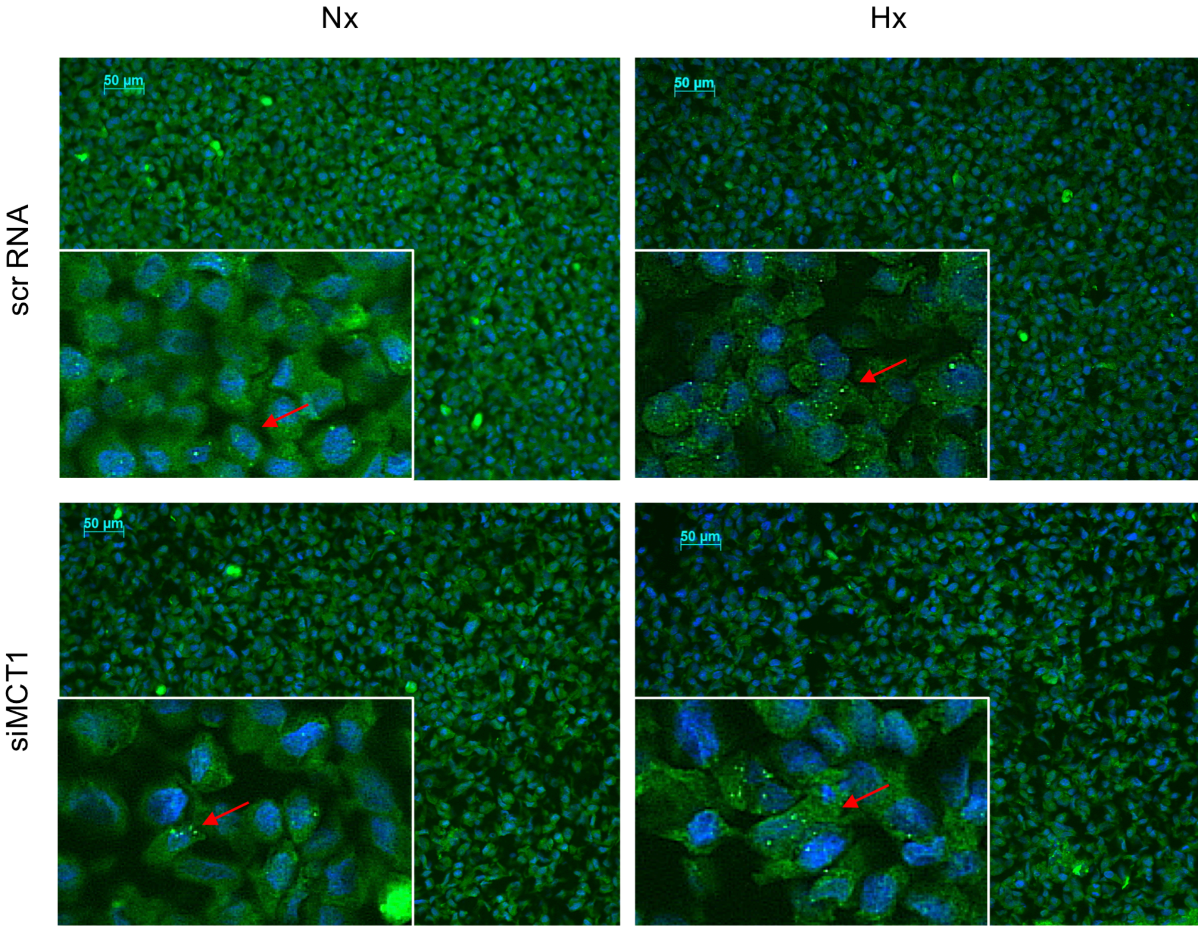

S5 Fig. Canine MCT1 staining. In situ hybridization staining with RNAscope® technology of specific MCT expression of canine MCT1. 17CM98 canine oral melanoma cells were transfected with siRNA against canine MCT1 using Lipofectamine 2000. Following transfection, cells were incubated in air (Nx) or 0.2% O_2 (Hx) for 16h. Positive signals appeared as dots (red arrows). Cells (scr and siMCT1) exposed to hypoxic conditions showed an increased RNA expression of MCT1. In the cells with RNA-based knockdown of canine MCT1 a reduced staining could be observed in normoxic as well as hypoxic cells compared with scr RNA control. Scale bar = 50µm.

The RNAscope® ISH Kit was purchased from ACDBio Tech company. Using custom made probes targeting canine MCT1 and MCT4 the staining was performed according to manufactures instruction.
