# Supplementary material for: Targeting neovascularization and respiration of tumor grafts grown on chick embryo chorioallantoic membranes
Source: PLoS One. 2021 May 17;16(5):e0251765. doi: 10.1371/journal.pone.0251765 (PMC8128225; doi:10.1371/journal.pone.0251765)
Supplement: S6 Fig — (PDF) [file pone.0251765.s009.pdf]

S6 Fig

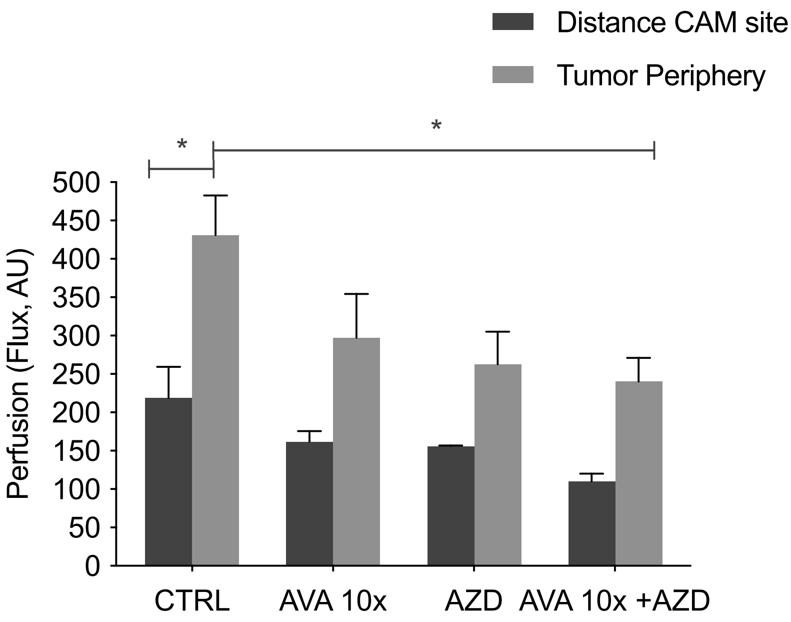

S6 Fig. Flux measurement on D17 tumor explants treated with 10-times higher AVA concentration. Laser-Speckle blood perfusion imaging of canine D17 osteosarcoma tumor explants. Quantified blood flow (Flux) measurements in D17 tumors (gray bars), relative to CAM-inherent background flux in tumor periphery, were recorded in response to the indicated treatments. Data means ± SEM, n=3. Statistical differences: one-was ANOVA, * = $p < 0.05$. AU = arbitrary units.
