# Supplementary material for: Targeting neovascularization and respiration of tumor grafts grown on chick embryo chorioallantoic membranes
Source: PLoS One. 2021 May 17;16(5):e0251765. doi: 10.1371/journal.pone.0251765 (PMC8128225; doi:10.1371/journal.pone.0251765)
Supplement: Attachment — Submitted filename: Response to Reviewers.pdf [file pone.0251765.s010.pdf]

Rebuttal letter

PONE-D-21-03224
Targeting neovascularization and lactate-fueled respiration in model tumors: assessment of anti-cancer treatment efficacy through chick embryo chorioallantoic membrane assays
PLOS ONE

Reviewer#1

1. *Since the paper is based on the use of CAM authors should explain better this assay as an extraembryonic membrane commonly used in vivo to study both new vessel formation and its inhibition in response to tissues, cells, or soluble factors (i.e. Ribatti D, Gualandris A, et al. J Vasc Res. 1997; Ribatti D, Vacca A et al. Curr Pharm Biotechnol. 2000; Ribatti D, Nico B, et al. Anat Rec. 2001).*

   Thank you for the comment. Introduction was revised and rewritten. The papers mentioned have been added to the reference list.

2. *In the Figure 5b, the specific primers used are lacking. In the same Figure, in the plots d and e the figure legends are missing. The authors should indicate this to make the figure understandable.*

   Primers used in Fig 5b are listed in S1 Table.

   We apologize that the legends to Fig 5d and e were not so easy visible. We now highlighted (bold) the letters “d” and “e” in the figure legend text for better visibility of the different panel legends of the figure.

3. *In the Figure 6, the figure legends are missing. The authors should add them.*

   Maybe the figure legends were overlooked because they were inside the manuscript text.

4. *In the Materials and Methods section the sources and relatives affiliations of several reagents are missing. The authors should complete the informations.*

   Thank you for this hint. We now added all sources and affiliations of the used reagents

5. *Please remove the abbreviations that are not necessary and introduce the missing abbreviations.*

   All unnecessary abbreviations were removed, and the missing abbreviations added

6. *All figures layout issues should be resolved in order to submit the paper for publication.*

   All figures were adjusted according to the comments above

Reviewer#2

1. *Reduce the "Introduction" paragraph, focusing on the main aspects and the purpose of the study*

   Thank your for the comment. Introduction paragraph was shortened and rewritten in order to focus on the main aspects

2. *Discussion: the part between 304-311 lines should be eliminated or merged with the introduction*

   The lines were deleted and included in the introduction

3. *Update the "References" section with more recent citations, if possible*

   Where possible references were updated

4. *Insert an "Abbreviations" section*

   Thank you for this hint. An "Abbreviations" section was included after the "Abstract"

5. *Figure 3: make the stairs uniform to the same unit*

   Figure 3 was adapted according to your suggestions.
